# Anti-inflammatory, immunomodulatory and anti-oxidant effects of *Ocimum basilicum *L*.* and its main constituents: A review

**DOI:** 10.22038/IJBMS.2023.67466.14783

**Published:** 2023

**Authors:** Elahe Kamelnia, Reza Mohebbati, Reyhane Kamelnia, Hesham R. El-Seedi, Mohammad Hossein Boskabady

**Affiliations:** 1 Department of Biology, Faculty of Science, Science and Research Branch, Islamic Azad University, Tehran, Iran; 2 Department of Physiology, Faculty of Medicine, Gonabad University of Medical Sciences, Gonabad, Iran; 3 Applied Biomedical Research Center, Mashhad University of Medical Sciences, Mashhad, Iran; 4 Institute of Biochemistry and Biophysics, University of Tehran, Tehran, Iran; 5 Department of Pharmaceutical Biosciences, Biomedical Center, Uppsala University, Uppsala, Sweden; 6 International Research Center for Food Nutrition and Safety, Jiangsu University, Zhenjiang 212013, China; 7 Department of Chemistry, Faculty of Science, Menoufia University, 32512 Shebin El-Kom, Egypt; 8 Department of Physiology, Faculty of Medicine, Mashhad University of Medical Sciences, Mashhad, Iran; # These authors contributed equally to this work

**Keywords:** Anti-oxidants, Basil extract, Inflammation, Immunomodulation, Ocimum basilicum

## Abstract

*Ocimum basilicum *L. (*O. basilicum*) is an ornamental and therapeutic plant with various pharmacological effects and medical applications. In this article, detailed information on the anti-oxidant, immunomodulatory, and anti-inﬂammatory properties of *O. basilicum* and its main constituents was provided. The literature survey of the different databases until the end of November 2021 was explored on the immunomodulatory, anti-inﬂammatory and anti-oxidant effects of the herb and its constituents. The plant and its constituents showed diverse pharmacological effects including immunomodulatory, anti-inflammatory and anti-oxidant properties by improving of the inflammatory mediators including interleukin (IL)-10, IL-4, tumor necrosis factor-alpha (TNF-α), interferon gamma (IFN-γ), nitric oxide (NO), serum levels of IFN-γ, IL10 and IL-4, Ig. G, Ig. M and phospholipase A2 (PLA2), immunoglobulin E (Ig. E), total protein (TP), oxidant and anti-oxidant markers. *O. basilicum* and its main constituents therefore, could be effective on the treatment of diseases associated with inflammation, immune dysregulation and oxidative stress. The present review article provides readers with organized information about the anti-oxidant, immunomodulatory, and anti-inﬂammatory properties of *O. basilicum*.

## Introduction

**Figure 1:**
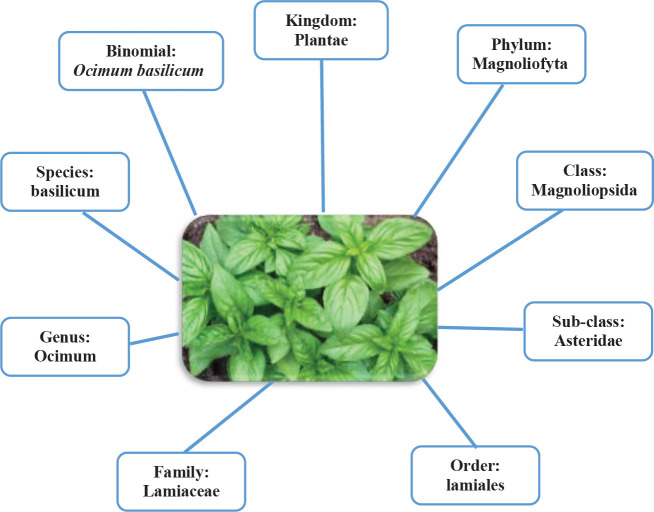
Taxonomical Hierarchy of the *Ocimum basilicum*

**Figure 2:**
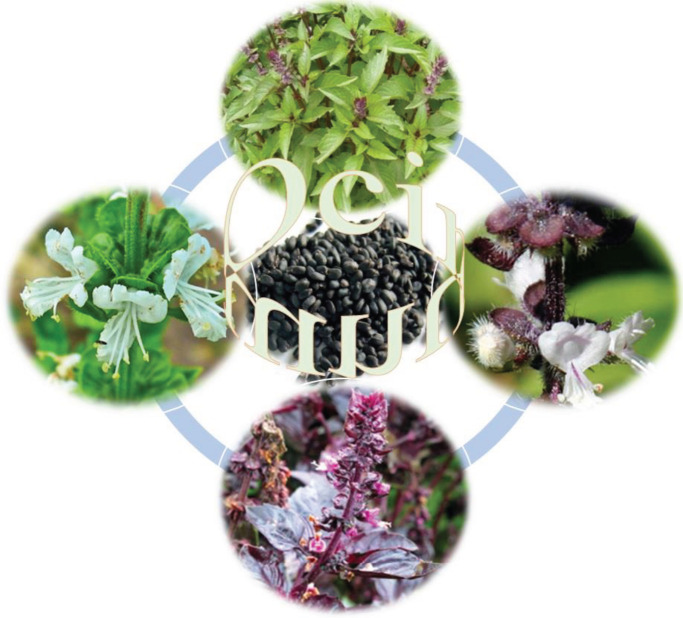
Flowers, leaves and seeds of *Ocimim basilicum*

The use of the natural materials of herbs by humans for diverse aims such as treatment of different diseases could be come back to the cave dwelling era. Traditional plants exert important role in discovery of new drugs. Medicinal plants, namely herbal medicine, are the most of bio resource of drugs for modern and traditional medicines, food supplements, nutraceuticals, folk medicines, and pharmaceutical intermediates. It was shown that out of 3,000 known plant species in the world, only 15% of them are used for medicine potential (1, 2). Recognizing the importance of plants in discovering new and safer therapeutic agents, screening of medicinal plants in terms of medicinal activities and phytochemical compounds is one of the active research fields worldwide (3). and several plants have been broadly used for their pharmacology and medicinal aspects (4). A plant contains a plenty of various molecules that may act synergistically on targeted elements of the complex cellular pathway so plants are essential source for medicinal compositions (5). Aromatic plants have been used for home treatment, subsistence, and traditional therapies and are used for local livelihoods and income generation (6). These plants have many applications at different fields such as food flavoring industries, cosmetic industries, alcoholic beverages, soft drinks, and pharmaceutical industries. Herbs as aromatic plants contain phytonutrients which can be used as a flavoring foods or beverage and most importantly used in the treatment of many diseases (7). The *Ocimum basilicum *L. (*O. basilicum*) is a plentiful source of polyphenolics and represents high diversity (8). The genus *Ocimum* belongs to the family Lamiaceae and various species of *Ocimum* are known that are used to treat various types of illnesses from old time, chiefly the species *O. basilicum* (Taxonomical Hierarchy of *O. basilicum* are shown in Figure 1). The *O. basilicum* is one of the most used plant that generally known as Sweet Basil which is found in tropical and subtropical regions of Africa, Asia, and south America (9). The *O. basilicum* leaves may taste like anise, via a strong, pungent, and sweet smell. The flowers of this plant are small, purple/white in color and it is generally removed to increase leaf yield (10, 11) (Figure 2). The fresh *O. basilicum* is usually used in cooking recipes and the plant species of this genus have many potentials in medical application. Nowadays, the interest in bioactive compounds of the Basil (*O. basilicum*) has increased due to its long-standing usage in folk medicine, especially anti-inflammatory, immunomodulatory, anti-oxidative, and anti-microbial properties. The *O. basilicum* is a herb that has been used in traditional Indian and Asian medicine for thousands years as a natural antibiotic, anti-inflammatory, analgesic, and diuretic (12, 13). Due to its chemical composition, it is used in cooking, perfumery, toothpaste, food industry, production of cosmetics and medicine (14). Several studies were focused their attention on this plant which is native in all part of the Mediterranean and widely commercially planted in Iran. The biological activity of *O. basilicum*, characterized it in various pharmacology, traditional and medicinal usage (15). This plant contains a wide range of natural products including polyphenols such as anthocyanins and flavonoids (16). This review article aimed to optimize and validate the phytochemical study and the anti-inflammatory, immunomodulatory, anti-inflammatory, and anti-oxidant effects of *O. basilicum* and its main constituents.

## Materials and Methods

***Methods***

The online publications were checked using variant search engine such as Google scholar, Scopus, PubMed, Web of Science, and Science direct. The main keywords used for searching were: *Ocimum basilicum*, sweet basil, linalool, eugenol, rosmarinic acid, polyphenol, flavonoid, essential oil, anti-oxidant, anti-inﬂammatory, and immunomodulatory in addition to related keywords alone and combined with each other. The published papers from 1998 to end of November 2021 in English language were included in the review.

## Results

***Phytochemical overview***

Bioactive compounds like polyphenols that found in vegetables and fruits cause their flavor, color, and pharmacological activities (17). The effects of *O. basilicum* in improvement of many diseases are related to its polyphenol and aromatic compounds. These compounds showed anti-oxidant, anti-allergic, anti-inflammatory, immunomodulatory and anti-viral properties. There are compositional variability in *O. basilicum* essential oils due to variations in geographic, climate, and agricultural conditions (18). Phenylpropanoids (methyl chavicol, methyl eugenol, eugenol, methyl cinnamate), monoterpenoids (1,8-cineole, linalool, citral, camphor, thymol, geraniol, ocimenes), and sesquiterpenoids (β-caryophyllene, β-elemene, trans-α- bergamotene, β-bisabolene, (E)-α-bisabolene,) are the usual ingredients that found in the *O. basilicum* essential oils (19). This plant contains about twenty ingredients such as estragole, linalool, depending on the species and cultivar. Methyl eugenol, 1, 8-cineole has been recognized by GC-MS (20), (Table 2). Chromatographic analysis of *O. basilicum* was shown a total of forty-nine ingredients accounting for the 98.8% of the total composition of the plant. Methyl chavicol was the main ingredient (74.9%) followed by α-bisabolene (1.1%) and linalool (18.4%) (21). According to the some researches, the essential oil composition of this plant was linalool (12.63%), eucalyptol (1.79%), eugenol (19.22%), α-bergamotene (3.96%), germacrene D (8.55%), α-terpineol (0.95%), α-guaiene (2.33%), camphor (0.70%), β-elemene (2.68%), tau-cadinol (15.13%), β-cariophylene (0.61%), α-copaene (0.33%), cubenol (1.78%), bornil acetate (1.97%), elixen (2.59%), metil eugenol (0.76%), epibiciclosesquiphelandrene (0.76%), δ-gurjunene (5.49%) β-farnesene (0.58%), α- cariophylene (1.67%), β cadinene (0.80%), α-bisabolol (0.35%), tau muralol (0.96%), and δ-cadinene (5.04%) (22). Polyphenols are found in the plants as glycosides esters or free aglycones. The hydroxybenzoic acid compounds are mostly available in the glucosides form while glucose esters of p-hydroxybenzoic, vanillic, and syringic acids have been found only sometimes (23, 24). They are classified pursuant to their chemical structures into flavonoids such as flavonols, flavones, isoflavones, neoflavonoids, chalcones, anthocyanidins, pro-anthocyanidins, and nonflavonoids, such as stilbenoids, phenolic acids, and phenolic amides. Elemental analysis and phytochemical screening of aqueous extract of this plant showed the presence of saponins, tannins, and cardiac glycosides and also elements such as calcium, potassium, sodium and magnesium. Phenolic compounds have competence properties like anti-oxidants acting as hydrogen donors, reducing agent, and radical oxygen quenchers (25). This data showed that *O. basilicum* contains minerals and bioactive compounds with various beneficial effects on health (26). Some results showed that green leaves of *O. basilicum* contain high concentration of minerals, vitamins , and oils (27). The essential oil of the flowers of this plant contain small amount of estragol, eucalyptol, ocimene, linalool, acetate, eugenol, 1-epibicyclosesquiphellandrene, menthone, methanol, cyclohexanol, cyclohexanone, nerol, and myrcenol (28). A research showed that the seeds of *O. basilicum* contain a gum which have major fractions of glucomannan with (1⟶4)-linked xylan and a minor fraction of glucan (29). Some example of the *O. basilicum* essential oil chemical ingredients in different parts are shown in Table 1 and the chemical structures of some compound are displayed in Table 2.

***Anti-inflammatory effect***

Inflammatory cells such as mast cells, T lymphocytes, and eosinophils have a main role in the pathogenesis of immune-mediated diseases (30, 31). Most of the human population is getting affected by inflammation related disorders. Therefore, anti-inflammatory agents could be of therapeutic value in the treatment of inflammatory diseases. The anti-inflammatory property of *O. basilicum* and its derivatives was shown in several studies.

***Anti-inflammatory effect of O. basilicum extracts and essential oil***

The anti-inflammatory properties of the *O. basilicum* essential oil complexed with β – cyclodextrin (OBEO/β-CD) in mice models of paw edema induced by carrageenan showed that conjugation of β- cyclodextrin with *O. basilicum* inhibited the number of total lymphocytes, leukocytes, granulocytes and monocytes in the abdominal cavity of carrageenan-induced animals, indicating the effectiveness of this complex in regulating leukocyte recruitment during an acute inflammatory response. The findings suggest that this plant can be used for the production of an anti-inflammatory drug (32). Various chemical elicitors such as jasmonic acid (JA), arachidonic acid (AA), and baminobutyricacid (BABA) can cause changes in the phenolic levels a then the anti-inflammatory potential of purple *O. basilicum* leaves. The results showed all tested elicitors increase the amounts of phenolic ingredients including phenolic acids and flavonoids and cause the highest anti-inflammatory activities in comparison with control group. Anthocyanin contents in *O. basilicum* is about 0.1 mg/g. The level of anthocyanins in its leaves has been significantly enhanced after stimulation with abiotic elicitors such as jasmonic acid, arachidonic acid and b-aminobutyric acid. Anthocyanins have ability to inhibition of lipoxygenase (LOX) activity and act as anti-inflammatory agents. The stimulation of basil by all of the elicitors enhanced its ability to inhibit the LOX activity, expressed as an IC_50_ value. LOX is responsible for the metabolism of the fatty acids and their metabolites eliciting inflammatory responses in the body (33). The analgesic effects of the *O. basilicum* essential oil in the inflammatory pain models in mice indicated that the analgesic effects of essential oil of the* O. basilicum* is similar to the linalool and eugenol which were mediated by mu- and delta -opioid pathways and further express that *O. basilicum* essential oil has a potential use as an analgesic agent for the alleviation of inflammatory pain (34). The anti-inflammatory effect of the *O. basilicum* extract against inflammation induced by adipocyte, possibly through suppression of Tnfrsf9 (TNF receptor superfamily member) has been shown (35). High *O. basilicum* extract concentration also decreased total WBC count and significantly increased anti-inflammatory and anti-oxidant parameters in the asthmatic rats similar to the dexamethasone (36). In a study, the chemical composition and systemic anti-inflammatory activity of the *O. basilicum* essential oil was investigated and verified via acute and chronic *in vivo *experiments as peritonitis, paw edema, vascular permeability, and granulomatous inflammation model by the participation of histamine and arachidonic acid pathways. The results demonstrate that the essential oil has more efficacious in the acute and chronic anti-inflammatory action. This research approves the therapeutic potential of *O. basilicum* and reinforces the validity of its use in the general medicine (37). In another study, the inhibitory effect of the *O. basilicum* extract on the key pro-inflammatory mediators and cytokines have been shown, which accounts for its anti-inflammatory effect (38, 39).

The extracts of the *O. basilicum* decreased the expression of inflammatory cytokine mRNA induced by co-culture, including those of IL-1β (Il1b), IL-6 (Il6), tumor necrosis factor-α (TNF-α), and CCL2 (Ccl2) and also suppressed the mRNA expression of NF-κB (Nfκb1) (35).

***Anti-inflammatory effect of O. basilicum constituents***

It was shown that mucilage of this plant could apply more protection against inflammatory mediators and oxidative stress in colitis suggesting this agent as a good candidate for colitis treatment as complementary therapy. Anti-inflammatory effect on colitis might be attributed to the presence of terpenoids and flavonoids which are known to inhibit the inflammatory signaling through NF-kB suppression. NF-kB through TNF-alpha activation has a pivotal role in the IBD pathology and recurrence and as its suppression could result in colitis remission both in the experimental and clinical settings. Besides, it revealed that treatment with this plant decrease the wet weight of distal colon segments and major damage score in comparison with the control which is well correlated with regression in local inflammation scores (40). In another study, the major ingredients of the residue fraction of *O. basilicum* were shown as methyl eugenol (11.35%), estragole (17.06%) and linoleic acid (11.40%), while the distillate fraction primarily contained a-cadinol (16.24%), methyl eugenol (16.96%) and a-bergamotene (11.92%). The distillate fraction of *O. basilicum* distinctly suppressed the generation of cytokines (IL-b, IL-6, TNF-a) and their gene expression in the LPS-induced Raw 264.7 cells and also suppressed iNOS and NO in *in vitro* model when compared with the oil. *O. basilicum* compounds can be an important source of natural anti-inflammatory agents after molecular distillation (41). Anti-inflammatory properties of the *O. basilicum* and its constituents are indicated in Table 3.

***Anti-oxidant effect***

Living cells of the organisms are involve in oxidative reactions for many goals including metabolism, cell communication, death or renewal, and defense mechanisms (42). Oxidizing agents and free radicals containing one or more unpaired electrons in their outer orbit that makes them reactive species namely electrophiles. The reactions of these free radicals with the organisms causes damage of the cell and tissue. The free radicals act on cellular components by oxidizing proteins, lipids, carbohydrates, and nucleic acids. The free radicals oxidized cellular components such as proteins, lipids, carbohydrates, and nucleic acids (43, 44). Anti-oxidants have a significant role in hampering a diversity of lifestyle-related diseases and aging so these are associated with active oxygen and lipid per-oxidation (45, 46). Herbal medicines applicate their anti-oxidant effects by multiple mechanisms (47-50). 

***Anti-oxidant effect of O. basilicum extracts and essential oil***

The anti-oxidant activities of the herbal extracts is mainly due to their capacity to be donors of hydrogen or electrons and to capture the free radicals (51). In cultivars using online acidic potassium permanganate chemiluminescence total phenolics, anti-oxidant content, and individual polyphenolics of the extracts from *O. basilicum* were examined. The results showed high polyphenolic producing in Red Rubin, Holy Green, and Basil Genovese low amount in Subja. Therefore, anti-oxidant potential was more potent in the purple flowered plant than its white flowered type (12). It was indicated that the extract of the *O. basilicum* acts as an anti-oxidant (52, 53) and effectively subjugates the effects of high oxidizing agents such as hydrogen peroxide (54, 55). These actions are attributed to its composition, which is rich in flavonoids and polyphenols as well as compounds such as rosmarinic acid (RA), all of which have well-known to show anti-oxidant property (43). The effects of the *O. basilicum* extract and its constituent, rosmarinic acid in the ovalbumin (OVA)-sensitized rats showed that treatment of the sensitized animals with all *O. basilicum* extract concentrations lead to significant decline in NO_2_, NO_3_ concentrations and total WBC count and also treatment with its two higher doses (1.5 and 3.0 mg/ml) cause decrease in malondialdehyde (MDA) value and monocytes percentage but increase catalase (CAT) and thiol values (56). In fact, the *O. basilicum* extract can be protected LDL from oxidation. Ethanolic extract of the *O. basilicum* has capability to decrease foam cell formation via reduction of the cholesterol synthesis and regulation of the activity of surface scavenger receptors (57). The anti-oxidant effect of the *O. basilicum* is beneficial to protect tissue and reducing carcinogenic effect of the electromagnetic field and might also provide protection of the ovary against reactive oxygen spaces (ROS). Exposure to 50 Hz of Electromagnetic fields (EMF) cause a significant enhance in the apoptotic granulosa cell percentages while *O. basilicum* extract significantly decrease the apoptotic granulosa cells. Therefore, *O. basilicum* extract could be considered as an anti-oxidant therapy against EMF exposure in the industrial area (58). Various extract of *O. basilicum* including n-BuOH, EtOAc and H_2_O extracts showed potent scavenger activity and notable inhibition of lipid peroxidation (LPx) in liposomes. Furthermore, CHCl3 and Et2O extracts indicated weaker effect in the neutralization of 2,2-diphenyl-1-picrylhydrazyl (DPPH), nitric oxide (NO), hydroxyl (OH) radicals, superoxide anion (O_2_ •−) and hydrogen peroxide (H_2_O_2_). The effect of the extracts on generation of the OH radicals and inhibition of LPx, CHCl3 and Et2O indicated a weak prooxidative properties of the herb (59). 

The anti-oxidant potential of methanolic extract of *O. basillicum* was studied using DPPH assay (60). Intake of the *O. basilicum* extract may decrease hepato-renal toxicity induced by acetaminophen (61) that attributed to its anti-oxidant property and may be due to suppression of liver lipid synthesis. *O. basilicum* administration modulated both kidney and liver pathological changes confirming the protective role of *O. basilicum* against hepatic/nephron toxicity and oxidative injury caused by paracetamol overdose (62). The better anti-inflammatory and anti-oxidant potential of the combination of essential oils of the *O. basilicum*/*O. gratissimum* occurs by inhibition of all isoforms of the cyclooxygenase by the inhibition of ≥ 98% for cyclooxygenase 1 and ≥ 67% for cyclooxygenase 2 (63). The essential oils of the *O. basilicum* indicated anti-oxidant and free radical-scavenging activities. In a study, the effect of growing season on chemical composition, antimicrobial and anti-oxidant activities of the essential oils from *O. basilicum* was investigated. The results revealed that in winter oxygenated monoterpenes were found to be richer, while those of summer were higher in sesquiterpene hydrocarbons. Linalool, the major component of the *O. basilicum* essential oil, exhibited lower anti-oxidant activity than the entire oil. The anti-oxidant activity of essential oils of the *O. basilicum* also might be attributed to the presence of other phenolic compounds (64). Essential oils of Iraqi growing *O. basilicum* contained linalool (48.69%), trans-α-bergamotene (8.23%), 1,8-cineole (14.00%) and eugenol (6.64%). The anti-oxidant investigation displayed strong inhibition of 110.8% against autoxidation by linoleic acid, while the scavenging of the DPPH radical gave a value of IC50 145.35 μg/mL. The findings showed that these essential oils could be used for pharmaceutical works and preservative in the food industry (65).

The use of the *O. basilicum* leaves powder as broilers chicken feed additive can ameliorate the anti-oxidant activity of broilers dose dependently so that its high concentrations causes increasing the serum CAT enzyme and reducing MDA levels (66). Improvement effect of *O. basilicum* essential oil on acetic acid–induced colitis in rats was investigated. Higher concentrations of *O. basilicum* essential oil (200 and 400 mL/kg) significantly reduced severity, area, and index of ulcer. Treatment with the essential oil of this plant also decreased the level of myeloperoxidase in colitis. These findings suggest that *O. basilicum* exhibits protective effect against acetic acid–induced colitis (67). A study was shown the anti-platelet aggregation effect of aqueous extract of *O. basilicum*. The plant extract suppressed the elevated vascular contractions induced by HCD and inhibited ADP-induced platelet aggregation. Thrombin-induced platelet activation was decreased by 15%, 23%, 40%, 38.4%, and 42% at the same doses of the extract. In hypercholesterolemia, one of the main causes of the lowered function of endothelial cells can be an enhance of superoxide release. Fatty acid and triglyceride-rich emulsions can stimulate leukocytes to generate ROS which are very toxic against the vascular wall cells (68)W¦X.

***Anti-oxidant effect of O. basilicum constituents***

Different parts of the *O. basilicum* like flowers, leaves and roots were found to be rich reserves of the anti-oxidant compounds. Anti-oxidant activity could be varied by the levels of flavonoids and phenols in *O. basilicum* (69). The polyphenols such as chicoric, rosmarinic, m-coumaric, caffeic, p-coumaric, and ferulic acids content of this plant exhibited anti-oxidant activity (70). The effects of the Rosmarinus acid (RA) in the ovalbumin sensitized rats showed that treatment of the sensitized animals with RA decreased cholesterol synthesis and lipid accumulation in human macrophages (56). Various therapeutic effects have been explained for RA such as anti-inflammatory, analgesic, immunomodulatory actions, and antibacterial activities. In a study, the effect of RA on inhibition of gentamicin-induced nephrotoxicity in rats and showed that RA reduced gentamicin nephrotoxicity via anti-oxidant activity by increase the renal GSH content and other anti-oxidant enzymes activities (71). Eugenol, carvacrol, thymol, and 4-allylphenol displayed better anti-oxidant activities than the other components of the plant. These components inhibited the oxidation of hexanal approximately 100% for 30 days and a concentration of 5μg/ml (72). In another research, the effect of RA on lung inflammatory cells, tracheal responsiveness (TR), and oxidant markers in the sensitized rats compared to the dexamethasone were evaluated and data indicated the dose-dependent effect of RA on tracheal responsiveness, inflammatory and oxidant-anti-oxidant parameters. Treated with this plant showed that anti-oxidant biomarkers (thiol, SOD and CAT) and percentage of lymphocyte were significantly less than those of control (56).

Anti-oxidant effects of the *O. basilicum* and its constituents are shown in Table 4.

***Immunomodulatory effect***

Immune system dysfunction can cause serious diseases. Immunomodulatory agents are able to improve and overcome many barriers to treatment methods, such as off-target side effects, inadequate immune stimulation, and bioactivity loss of immune agent. Immunotherapy means treating a disease by suppressing or activating immune system (73). Immune pathways driven by T-helper 2 (Th2) cells which produce IL-5, IL-4, and IL-13 can involve in the pathophysiology of many inflammatory diseases. The imbalance of Th1/Th2 toward increased Th2 activity was shown in the pathogenesis of allergies (9, 74). Th2 cells release cytokines such as IL- 4 and IL- 5 that leads to the production of IgE by B cells and therefore they have an important role in allergy. In fact, IL-4 causes excessive production of immunoglobulin E (IgE), IL-5, activates eosinophils, and IL-13 which lead to mucus hyper-secretion (75-77). Immune and inflammatory cells such as dendritic cells, leukocytes, and T cells, as well as cytokines, play important roles in immune reactions (78). Flavonoids, triterpenes, and catechins are biologically active components of the plants that play a main role in immunoregulatory activity (79).

***Immunomodulatory effect of O. basilicum extracts and essential oil***

Hydro-ethanolic extract of *O. basilicum* showed the anti-inflammatory effects on lung pathological changes in OVA-induced asthma. Treatment with *O. basilicum* extract lead to decrease IgE, IL-4, phospholipase A2 (PLA2) and total protein (TP) levels but increase interferon gamma (IFN-γ) and IFN-γ/IL-4 ratio in comparison with the healthy rats. The plant significantly improved the pathological changes of the sensitized rats (80). Also, in the similar model, *O. basilicum *extract decreased total WBC count, percentages of monocytes, eosinophils, neutrophils, and levels of oxidant markers compared to the untreated sensitized rats (36). In another study, the anti-inflammatory activity of the *O. basilicum* extracts using peripheral blood mononuclear cells (PBMC) of the healthy individuals showed that methanolic extract of the *O. basilicum* inhibits the key pro-inflammatory cytokines like IL-1β, IL-2, TNF-α, and other mediators, which accounts for its anti-inflammatory effect (38). The methanolic extract from this plant can inhibits platelet aggregation induced by collagen and it has the most potent inhibitory effects on platelet aggregation induced by ADP (81) and also showed inhibitory activity to counter HIV-1 reverse transcriptase (82).

The immune effects of *O. basilicum* have been reported previously (83). Farida *et al.* showed that administration of *O. basilicum* leaves powder as a feed additive in chicken can boost the innate and adaptive immune response to Newcastle disease virus (NDV) vaccine (66). The effect of *O. basilicum* oil on the non-specific immune response of Nile-tilapia (Oreochromis Nilotic us) and resistance against some aquaculture disease such as Aeromonas infection was also demonstrated (84). The direct immunomodulatory effect of extract of the *O. basilicum* on human immune cells by Th2 and Th1 cells as well as regulatory T derived cytokines, possibly via the ERK2 signal pathway was also reported. This plant could be used for treating immune dysregulatory disorders. Extract of the *O. basilicum* suppressed cytokines produced by Th1 (IL-2, TNF-β, and IFN-γ), Th2 (IL-10, IL-5) as well as regulatory T (TGF-β) cells, and expression of ERK2 mRNA in peripheral blood mononuclear cells (PBMC) and also suppressed some of the cytokines. Therefore, the results indicate that *O. basilicum* has direct immunomodulatory effect on basic functional properties of human immune cells, possibly mediated by the ERK2, MAP-kinase signal pathway (85). The extract of *O. basilicum* leaves on animal model of asthma increased the IFN-γ/IL-4 ratio (Th1/Th2 balance) but decreased bronchoalveolar lavage fluid (BALF) levels of IgE, phospholipases A2 (sPLA2), and TP (9). The effect of aqueous extract of the* O. basilicum* at 31.25, 62.5, 125 and 250 mg/ml concentrations on lymphocyte proliferation showed 36 and 80% decrease in proliferation of resident lymphocytes at 125 and 250 mg/ml respectively (86). In a study the cytoprotective effects of RA from *O. basilicum* against mycotoxin, aflatoxin, and ochratoxin was shown by dose dependently inhibition of DNA and protein synthesis. Apoptosis was prevented by decline of DNA fragmentation and inhibition of caspase-3 (87). Ethanolic extract of *O. basilicum* and earning flavonoid content at 400 mg/kg/day in mice showed an increase in circulating antibody titer in response to sheep red blood cells (SRBCs) and also increased the percentage neutrophils adhesion to nylon fibers and phagocytic activity (88).

In another study, *O. basilicum* extracts, reduced the expression of inflammatory cytokine mRNA, including IL-1β (Il1b), IL-6 (Il6), TNF-α, and CCL2 and suppresses the mRNA expression of NF-κB (Nfκb1), a transcription factor of inflammatory cytokines, and Tnfrsf9 expression (89).

***Immunomodulatory effect of O. basilicum constituents***

Compounds extracted from *O. basilicum* showed immunomodulatory action happening in the cellular level. In a study, it has been shown that constituent of *O. basilicum* reduced IL-4, IgE, PLA2 and TP levels, but enhanced IFN-γ/IL-4 ratio and therefore affected lung pathological changes (80). The flavonoid content of this plant can produce a marked increase in percentage neutrophils adhesion to nylon fibers and phagocytic activity and show immunostimulant effect (89). Immunomodulatory effect of *O. basilicum* and its constituents are summarized in Table 5.

**Table 1:** Some examples of chemical composition of *Ocimum basilicum* essential oil in various parts of plant

**Part of plant**	**Major constituent**	**%**	**Main effect**	**Reference**
Seed	Linalool	31.6%	Antioxidant and antimicrobial	
	Chavicol	23.8 %		
Leave	Linalool	52.1%	Antioxidant and antimicrobial	(91)
	Linalyl acetate	19.1%		
Leave	Methylchavico	47%	Anti-oxidant	(92)
	Geranial	19%		
	Neral	15%		
Leave	Methyl eugenol	42.18%	Antifungal and antiaflatoxin of plant	(93)
	Eugenol	4.89%		
	1,8-cineole	4.88%		
	B-caryophyllene	4.37%		
Leave	Estragole	87.869%	Antibiotics	(94)
	Cadinol	2.922%		
	α-Bergamotene	2.770%		
	τ-Linalool	1.347%		
Seed	Linalool	35.99%	Antibacterial	(95)
	1,8-cineole	22.91%		
	p-Cymene	35.5%		
Leaves	Linalool	29.23%	Insecticidal activity	(96)
	Methyl cinnamate	18.97%		
	Eugenol	5.84%		
Flowers	Linalool	72.3%	Sweet-spicy odor	(97)
	Methyl chavicol	19.5%		
Leaves and stems	Eugenol	42.74%	Biological activities	(98)
	Linalool	20.54%		
	Eucalyptol	15.27%		
Leaves	Estragole	55.95%	Antifungal	(99)
	1,8-Cineole	10.56%		
	Methyl eugenol	10.09%		
	Linalool	5.57%		
Overground part	Methyl eugenol	78.02%	Anti-oxidant	(100)
	α-cubebene	6.17%		
	Nerol	0.83%		
	ε-muurolene	0.74%		
Leaves	β-Guaiene	16.89%	Antibacterial, antioxidant and larvicidal activities	(101)
	Cadinol	15.66%		
	Nona-2, 4, 6-triene	11.36%		
	Phytol	11.68%		
Seeds	Eugenol	50.8%	Promote metabolite production and stress resistance	(102)
	Linalool	54.7%		
Leaves, flower, stems	4,7 Dimethoxy-1-indanone	21.73%	Anti-diabetic activity	(103)
	Palmitic acid	7.60%		
Leaves	Methyl-chavicol	52.4%	antinociceptive effects	(104)

**Table 2:** Chemical structure of the most important compounds of *Ocimum basilicum*

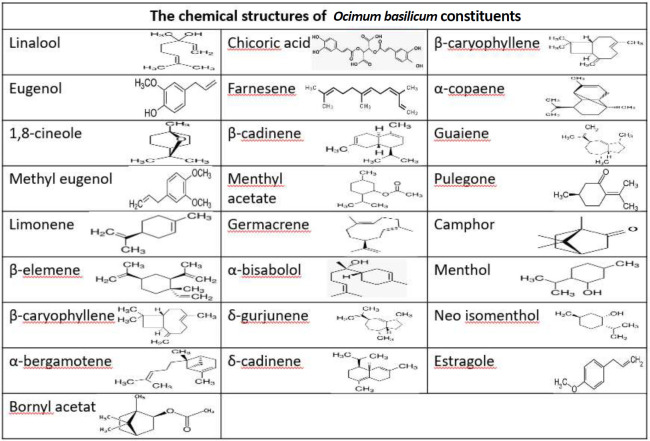

**Table 3:** Anti-inflammatory effects of *Ocimum basilicum* and its constituents

Preparation	Part of Plant	Dose	Experimental design	Effect	Ref.
**Essential oils**	Leaves	315–500 μM	DPPH assay	Decreased MDA, increased thiol, SOD and CAT	(105)
**Ethanolic E.**	Leaves and seed	0.01–1 mg/mL	LPS-stimulated RAW 264.7 macrophage cells	Prevented pathological inflammation, reduced NO	(106)
**Rosmarinic acid**	Leaves	0.250 and 0.500 mg/mL	Ovalbumin- induced asthma	Improved tracheal responsiveness	(56)
**Estragole and linalool**	Flowers and leaves	0.27–0.37 µg/mL	Alpha-Amylase and Lipase Enzymes-stimulated ISMD	PPA and PPL inhibitory activities	(107)
**Ethanolic acid**	Leaves	0.75, 1.50 and 3.00 mg/mL	Ovalbumin- induced asthma	Decreased tracheal responses and lung inflammatory cells	(36)
**Rosmarinic** **Acid**	Leaves	0.125, 0.250 and 0.500 mg/mL	Ovalbumin- induced asthma	Decreased tracheal responses and lung inflammatory cells	(108)
**Essential oils**	Whole plant	100 or 200 mg/kg	Pirin-induced gastric ulcer	prophylactic effects on aspirin-induced gastric ulcers	(109)
**Ethanolic E.**	Aerial parts	100, 300 and 500 mg/kg	Cisplatin-induced acute renal injury	Nephroprotective activity	(110)
**Phenolic**	Leaves	2.5, 5, 10 mg/kg	Carrageenan induced-paw inflammation	Decreased inflammatory reaction	(111)
**Phenolic**	Seed	Estragole and linalool (AgNO3: 5, 25 μM as 3.30, 4.37 μg/g DW)	CdCl2, AgNO3 and YE-incubated cells	Increasing pharmaceutical active ingredients	(112)

Ref.: Reference; EO: Essential Oil; PPA: Porcine pancreatic α-amylase; PPL: Lipase; CdCl_2_: Cadmium chloride; AgNO_3_: Silver nitrate; YE: Yeast extract; DPPH: (2,2-diphenyl-1-picrylhydrazyl) assay; ISMD: In silico molecular docking

**Table 4:** Anti-oxidant effects of *Ocimum basilicum* and its constituents

Preparation	Part of Plant	Dose	Experimental design	Effect	Reference
**Phenolic compounds**	Leaves	705.0 and 596.5 µg/g	DPPH assay	Improved production of phenolic compounds as antioxidant	(33)
**Essential oils**	Stems and leaves	0.4 g/mL and 5.4 g/mL	DPPH assay	Inhibited linoleic acid oxidation and peroxidation	(64)
**Phenolic**	Leaves	64.71, 96.42 mg/mL	DPPH assay	Protected human from food-borne pathogens	(113)
**Essential oils**	-	-	simplex-lattice design using GC/MS analysis	Highest antioxidant activity was obtained for a 75:8:17 percentage composition mixture for marjoram, basil and rosemary, respectively	(114)
**Phenolic**	Leaves	100 and 200 mg/kg,	IR-induced cerebral damage	Prevented stroke, restored GSH, attenuated short-term memory and motor coordination impairment	(115)
**Essential oil**	Aerial parts	200 and 400 mL/kg	Acetic acid–induced colitis	Decreased level of myeloperoxidase	(67)

IR: Ischemia and reperfusion; DPPH: (2,2-diphenyl-1-picrylhydrazyl) assay; GC/MS: Gas chromatography − mass spectrometry

**Table 5:** Immunomodulatory effect of *Ocimum basilicum* and its constituents

Preparation	Part of Plant	Dose	Experimental design	Effect	Reference
**Hydro-ethanolic E.**	Leaves	0.75, 1.50 and 3.00 mg/mL	Ovalbumin-induced asthma	Decreased IL-4, IgE, PLA2 and TP levels, but increased IFN-γ/IL-4 ratio	(9)
**Methanolic E.**	Whole plant	1 µg/mL	LPS-stimulated PBMC	Inhibited TNF-α, IL-1β, and IL-2	(38)
**Caffeic (CA) and p-coumaric acid**	Whole plant	0.135, 0.27, and 0.54 μg/ml of OB extract, 2.5, 5, and 10 μg/ml of CA, 5, 10, and 20 μg/ml of pCA	PBMC Lymphoproliferation test	Suppressed Th1 (IL-2, IFN-γ, and TNF-β), Th2 (IL-5, IL-10) cytokines and regulatory T (TGF-β) cells, and expressed ERK2 mRNA in PBMC	(85)
**Hydro-ethanolic E.**	Leaves	0.75, 1.50 and 3.00 mg/mL	Ovalbumin-induced rat model of asthma	Decreased IL-4, IgE, PLA2 and TP levels, but increased IFN-γ/IL-4 ratio	(9)
**Methanol and aqueous E.**	Leaves	3.9, 7.8, 15.6, 31.2, 62.5, 125 and 250 μg/ml	Lymphoproliferation test	Increased pool of lymphocyte	(86)
**Essential oils**	Leaves	Serial dilution 50 to 350 mg/mL	Anti-cancer and cytotoxicity activities	Obtained IC_50_ values were 90.5 and 96.3 µg mL−1,	(89)
**Hydro-ethanolic E. **	Leaves	0.75, 1.50, and 3.00 mg/ml	Ovalbumin-induced asthma	Decreased total WBC count, percentages of eosinophils, monocytes, neutrophils, and levels of oxidant biomarkers	(36)
**Aqueous and ethanolic E.**	Leaves	400 mg/kg/day	Cyclophosphamide and levamisole- induced immunosuppression	increased production of circulating antibody titer in response to SRBCs	(88)

PBMC: Peripheral blood mononuclear cells, TNF-α: Tumor necrosis factor-α, IL: Interleukin, SRBCs: Sheep red blood cells, WBC: White blood cell, ERK2: Extracellular signal-regulated kinase 2

## Discussion

The extensive survey of literature displayed that *O. basilicum* has an enormous spectrum of pharmacological activities. The current review is meant to describe the importance of *O. basilicum* in the field of herbal medication. The effect of *O. basilicum* and its constituents on inflammation, oxidative stress and immune system were reviewed. Crude extracts and essential oil of different parts of this plant have been used for their various effects like anti-inflammatory, immunomodulatory, anti-oxidant, due to its bioactive phytocomponents. According to the results of several studies using various models, this plant and its constituents such as polysaccharides, phenolics and flavonoids indicated anti-oxidant, anti-inflammatory, immunomodulatory and anti-microbial effects. *O. basilicum* and its constituents shows anti-inflammatory and immunomodulatory property via improvement of inflammatory cells and inflammatory markers including IL-4, IL-10, TNF-α, IFN-γ and other cytokines. Anti-oxidant effects of this plant and its constituents were also shown by scavenging free radicals, reduction of oxidant agents and increase of anti-oxidant parameters. *O. basilicum* and its constituents modulated immune system via improvement of T-lymphocytes, and NK cells as well as inflammatory and anti-inflammatory cytokines, and Th1/Th2 balance. The wide range of study indicates that it is very beneficial for the improvement of current drugs and more work can be done to explore its advantage therapeutic potential in inflammatory, oxidative stress and immune dis-regulatory disorders.

## Conclusion

The review articles showed anti-inflammatory, immunomodulatory and anti-oxidant effects of *O. basilicum* and its main constituents in various conditions which indicate possible therapeutic effects of the plant and its derivatives on inflammatory disorders, oxidative stress alignment and immune-dysregulatory diseases. However, more studies including clinical trials on the anti-inflammatory, immunomodulatory and anti-oxidant effects of* O. basilicum* and its components are needed to performed before the plant and its constituents could be used for clinical purposes.

## Authors’ Contributions

MHB designed the study; EK and RK collected data; HRE and RM discussed the results and strategy; MHB Supervised, directed and managed the study; MHB and RM Final approved of the version to be published. 

## Conflicts of Interest

None.

## References

[1] Osei Akoto C, Acheampong A, Boakye YD, Naazo AA, Adomah DH (2020). Anti-inflammatory, anti-oxidant, and anthelmintic activities of Ocimum basilicum (Sweet Basil) fruits. J Chem.

[2] Boskabady MH, Alitaneh S, Alavinezhad A (2014). Carum copticum A herbal medicine with various pharmacological effects. BioMed Res Int.

[3] Boskabadi J, Saadat S, Boskabady MH (20201). The relaxant effect of plantago major on rat tracheal smooth muscles and its possible mechanisms. Iran J Allergy Asthma Immunol.

[4] Kunwar RM, Mahat L, Acharya RP, Bussmann RW (2013). Medicinal plants, traditional medicine, markets and management in far-west Nepal. J Ethnobiol Ethnomed.

[5] Kumar S, Bajwa B, Kuldeep S, Kalia A (2013). Anti-inflammatory activity of herbal plants: A review. Int J Adv Pharm Biol Chem.

[6] Rokaya MB, Münzbergová Z, Timsina B (2010). Ethnobotanical study of medicinal plants from the Humla district of western Nepal. J Ethnopharmacol.

[7] Boskabady MH, Keyhanmanesh R, Khamneh S, Ebrahimi MA (2011). The effect of Nigella sativa extract on tracheal responsiveness and lung inflammation in ovalbumin-sensitized guinea pigs. Clinics..

[8] Shakeri F, Boskabady MH (2015). A review of the relaxant effect of various medicinal plants on tracheal smooth muscle, their possible mechanism (s) and potency. J Ethnopharmacol.

[9] Eftekhar N, Moghimi A, Roshan NM, Saadat S, Boskabady MH (2019). Immunomodulatory and anti-inflammatory effects of hydro-ethanolic extract of Ocimum basilicum leaves and its effect on lung pathological changes in an ovalbumin-induced rat model of asthma. BMC Complement Alternat Med.

[10] Shakeri F, Hosseini M, Ghorbani A (2019). Neuropharmacological effects of Ocimum basilicum and its constituents. Physiol Pharmacol.

[11] Hozayen WG, El-Desouky MA, Soliman HA, Ahmed RR, Khaliefa AK (2016). Anti-osteoporotic effect of Petroselinum crispum, Ocimum basilicum and Cichorium intybus L in glucocorticoid-induced osteoporosis in rats. BMC Complement Alternat Med.

[12] Srivastava S, Cahill DM, Conlan XA, Adholeya A (2014). A novel in vitro whole plant system for analysis of polyphenolics and their anti-oxidant potential in cultivars of Ocimum basilicum. J Agric Food Chem.

[13] Al Abbasy DW, Pathare N, Al-Sabahi JN, Khan SA (2015). Chemical composition and antibacterial activity of essential oil isolated from Omani basil (Ocimum basilicum Linn ). Asian Pacific J Trop Dis.

[14] da Silva Moura EdS, D’Antonino Faroni LR, Fernandes Heleno FF, Aparecida Zinato Rodrigues AAZ, Figueiredo Prates LH, Lopes Ribeiro de Queiroz ME (2020). Optimal extraction of Ocimum basilicum essential oil by association of ultrasound and hydrodistillation and its potential as a biopesticide against a major stored grains pest. Mol.

[15] Purushothaman B, Prasanna Srinivasan R, Suganthi P, Ranganathan B, Gimbun J, Shanmugam K (2018). A comprehensive review on Ocimum basilicum. J Nat Remed.

[16] Pirtarighat S, Ghannadnia M, Baghshahi S (2019). Biosynthesis of silver nanoparticles using Ocimum basilicum cultured under controlled conditions for bactericidal application. Materials Science and Engineering: C.

[17] Yahfoufi N, Alsadi N, Jambi M, Matar C (2018). The immunomodulatory and anti-inflammatory role of polyphenols. Nutrients.

[18] Verma RS, Padalia RC, Chauhan A, Thul ST (2013). Exploring compositional diversity in the essential oils of 34 Ocimum taxa from Indian flora. Ind Crops Prod.

[19] Padalia RC, Verma RS, Chauhan A (2014). Analyses of organ specific variations in essential oils of four Ocimum species. J Essential Oil Res.

[20] Radulović NS, Blagojević PD, Miltojević AB (2013). α-Linalool–a marker compound of forged/synthetic sweet basil (Ocimum basilicum L ) essential oils. J Sci Food Agric.

[21] Fitsiou E, Mitropoulou G, Spyridopoulou K, Tiptiri-Kourpeti A, Vamvakias M, Bardouki H (2016). Phytochemical profile and evaluation of the biological activities of essential oils derived from the Greek aromatic plant species Ocimum basilicum, mentha spicata, pimpinella anisum and fortunella margarita. Mol.

[22] Zamfirache M-M, Padurariu C, Burzo I, Olteanu Z, Boz I, Lamban C (2011). Research regarding the chemical composition of the volatile oil of some taxa belonging to the genus Ocimum. Analele Stiintifice ale Universitatii” Al I Cuza” din Iasi.

[23] Tsimogiannis D, Oreopoulou V Classification of phenolic compounds in plants. Polyphenols in plants: Elsevier.

[24] Tsao R (2010). Chemistry and biochemistry of dietary polyphenols. Nutrients.

[25] Sahin H, Turumtay EA, Yildiz O, Kolayli S (2015). Grayanotoxin-III detection and anti-oxidant activity of mad honey. Int J Food Properties.

[26] Daniel V, Daniang I, Nimyel N (2011). Phytochemical analysis and mineral elements composition of Ocimum basilicum obtained in JOS METROPOLIS, Plateau state Nigeria. IJET-IJENS.

[27] Khare CP ( 2008). Indian medicinal plants: an illustrated dictionary.

[28] Bihari CG, Behera M, Kumar JP, Kumar TS (2011). Pharmacognostical and phytochemical investigation of various tulsi plants available in south eastern Odisha. Int J Res Pharm Biomed Sci.

[29] Razavi SM, Mortazavi SA, Matia-Merino L, Hosseini-Parvar SH, Motamedzadegan A, Khanipour E (2009). Optimisation study of gum extraction from Basil seeds (Ocimum basilicum ). Int J Food Sci Technol.

[30] Shakeri F, Eftekhar N, Roshan NM, Rezaee R, Moghimi A, Boskabady M (2019). Rosmarinic acid affects immunological and inflammatory mediator levels and restores lung pathological features in asthmatic rats. Allergologia et Immunopathol.

[31] Boskabady MH, Jalali S (2013). Effect of carvacrol on tracheal responsiveness, inflammatory mediators, total and differential WBC count in blood of sensitized guinea pigs. Exp Biol Med.

[32] Rodrigues LB, Martins AOBPB, Ribeiro-Filho J, Cesário FRAS, e Castro FF, de Albuquerque TR (2017). Anti-inflammatory activity of the essential oil obtained from Ocimum basilicum complexed with β-cyclodextrin (β-CD) in mice. Food Chem Toxicol.

[33] Złotek U, Szymanowska U, Karaś M, Świeca M (2016). Anti-oxidative and anti-inflammatory potential of phenolics from purple basil (Ocimum basilicum L ) leaves induced by jasmonic, arachidonic and β-aminobutyric acid elicitation. Int J Food Sci Technol.

[34] Bae AH, Kim G, Seol GH, Lee SB, Lee JM, Chang W (2020). Delta-and mu-opioid pathways are involved in the analgesic effect of Ocimum basilicum L in mice. J Ethnopharmacol.

[35] Takeuchi H, Takahashi-Muto C, Nagase M, Kassai M, Tanaka-Yachi R, Kiyose C (2020). Anti-inflammatory effects of extracts of sweet basil (Ocimum basilicum L ) on a co-culture of 3T3-L1 Adipocytes and RAW264 7 macrophages. J Oleo Sci.

[36] Eftekhar N, Moghimi A, Hossein Boskabady M, Kaveh M, Shakeri F (2019). Ocimum basilicum affects tracheal responsiveness, lung inflammatory cells and oxidant–anti-oxidant biomarkers in sensitized rats. Drug Chem Toxicol.

[37] Rodrigues LB, Martins AOBPB, Cesário FRAS, e Castro FF, de Albuquerque TR, Fernandes MNM (2016). Anti-inflammatory and antiedematogenic activity of the Ocimum basilicum essential oil and its main compound estragole: In vivo mouse models. Chemico-biol Interact.

[38] Selvakkumar C, Gayathri B, Vinaykumar KS, Lakshmi BS, Balakrishnan A (2007). Potential anti-inflammatory properties of crude alcoholic extract of Ocimum basilicum L in human peripheral blood mononuclear cells. J Health Sci.

[39] Wibisono PAP (2021). Utilization of basil leaves (Ocimum basilicum) and avocado leaves (persea americana mill) as anti-inflammatory gel and antibiotics in myasis disease. Khazanah: Jurnal Mahasiswa.

[40] Saeidi F, Sajjadi SE, Minaiyan M (2018). Antiinflammatory effect of Ocimum basilicum Linn seeds hydroalcoholic extract and mucilage on acetic acid-induced colitis in rats. J Rep Pharm Sci.

[41] Li H, Ge Y, Luo Z, Zhou Y, Zhang X, Zhang J (2017). Evaluation of the chemical composition, anti-oxidant and anti-inflammatory activities of distillate and residue fractions of sweet basil essential oil. J Food Sci Technol.

[42] Ingram S, Diotallevi M (2017). Reactive oxygen species: Rapid fire in inflammation. Biochem.

[43] Güez CM, Souza ROd, Fischer P, Leão MFdM, Duarte JA, Boligon AA (2017). Evaluation of basil extract (Ocimum basilicum on oxidative, anti-genotoxic and anti-inflammatory effects in human leukocytes cell cultures exposed to challenging agents. Brazil J Pharm Sci.

[44] Shakeri F, Boskabady MH (2017). Anti-inflammatory, anti-oxidant, and immunomodulatory effects of curcumin in ovalbumin-sensitized rat. BioFactors.

[45] Paseban M, Mohebbati R, Niazmand S, Sathyapalan Th, Sahebkar AH (2019). Comparison of the neuroprotective effects of asperin, atorvastatin, captopril and metformin in diabetes mellitus. Biomolecules.

[46] Mohebbati R, Hosseini M, Haghshenas M, Nazariborun A, Beheshti F (2017). Th eeffects of Nigella sativa extract on renal tissue oxidative damage during neonatal and juvenile growth in propylthiouracil-induced hypothyroid rats. Endocr Regul.

[47] Yadav RD, Jain S, Alok S, Mahor A, Bharti JP, Jaiswal M (2011). Herbal plants used in the treatment of urolithiasis: A review. Int J Pharm Sci Res.

[48] Parhizgar S, Hosseinian S, Hadjzadeh MAR, Soukhtanloo M, Ebrahimzadeh A, Mohebbati R (2016). Renoprotective effect of Plantago major against nephrotoxicity and oxidative stress induced by cisplatin. Iran J Kidney Dis.

[49] Mohebbati R, Khazdair MR, Hedayati M (2017). Neuroprotective effects of medicinal plants and their constituents on different induced neurotoxicity methods: A review. J Rep Pharm Sci.

[50] Khazdair MR, Mohebbati R, Karimi S, Abbasnezhad A, Haghshenas M (2016). The protective effects of Curcuma longa extract on oxidative stress markers in the liver induced by Adriamycin in rats. Physiol Pharmacol.

[51] Tewari D, Pandey HK, Sah AN, Meena H, Chander V, Singh R (2015). Phytochemical, anti-oxidant and antidepressant evaluation of Ocimum basilicum enuiflorum kilimandscharicum grown in India. J Biol Active Products Nat.

[52] Alkadi H (2021). Ocimum basilicum: A candidate plant against aflatoxins production with anti-oxidant activity. J Mater Environ Sci.

[53] Chai PW (2021). Antibacterial and anti-oxidant activities of Cinnamon (Cinnamomum Zeylanicum), Ginger (Zingiber Officinale) and Sweet Basil (Ocimum basilicum) Essential Oils.

[54] Othman MS, Khaled AM, Al-Bagawi AH, Fareid MA, Ghany RA, Habotta OA (2021). Hepatorenal protective efficacy of flavonoids from Ocimum basilicum extract in diabetic albino rats: A focus on hypoglycemic, anti-oxidant, anti-inflammatory and anti-apoptotic activities. Biomed Pharmacother.

[55] Nguyen VT, Nguyen NQ, Thi NQN, Thi CQN, Truc TT, Nghi PTB (2021). Studies on chemical, polyphenol content, flavonoid content, and anti-oxidant activity of sweet basil leaves (Ocimum basilicum L.

[56] Eftekhar N, Moghimi A, Boskabady MH (2018). The effects of Ocimum basilicum extract and its constituent, rosmarinic acid on total and differential blood WBC, serum levels of NO, MDA, thiol, SOD, and CAT in ovalbumin sensitized rats. Iran J Pharm Res.

[57] Bravo E, Amrani S, Aziz M, Harnafi H, Napolitano M (2008). Ocimum basilicum ethanolic extract decreases cholesterol synthesis and lipid accumulation in human macrophages. Fitoterapia.

[58] Khaki A, Khaki AA, Ezzatzadeh A, Hamidreza A (2013). Effect of Ocimum basilicum on ovary tissue histopathology after exposure to electromagnetic fields (EMF) in rats. African J Pharm Pharmacol.

[59] Kaurinovic B, Popovic M, Vlaisavljevic S, Trivic S (2011). Anti-oxidant capacity of Ocimum basilicum and Origanum vulgare L extracts. Mol.

[60] James O, Eniola OJ, Nnacheta O (2008). Comparative evaluation of anti-oxidant capacity and cytotoxicity of two Nigerian Ocimum species. Am J Plant Sci.

[61] Teofilović B, Tomas A, Martić N, Stilinović N, Popović M, Čapo I (2021). Anti-oxidant and hepatoprotective potential of sweet basil (Ocimum basilicum extract in acetaminophen-induced hepatotoxicity in rats. J Funct Foods.

[62] Soliman A, Rizk M, Shalaby M, Elkomy A (2020). Mechanisms of hepato-renal protective activity of Ocimum basilicum leaf extract against paracetamol toxicity in rat model. Adv Anim Vet Sci.

[63] Fokou JBH, Pierre NJ, Gisele EL, Christian NC, Michel JDP, Laza IM (2020). In vitro anti-oxidant and anti-inflammatory potential of the optimized combinations of essential oils from three cameroon grew Ocimum L. J Pharm Pharmacol.

[64] Hussain AI, Anwar F, Sherazi STH, Przybylski R (2008). Chemical composition, anti-oxidant and antimicrobial activities of basil (Ocimum basilicum) essential oils depends on seasonal variations. Food Chem.

[65] Ahmed AS, Fanokh AKM, Mahdi MA (2019). Phytochemical identification and anti-oxidant study of essential oil constituents of ocimum basilicum l. Growing in Iraq. Pharmacog J.

[66] Mohamed FH, Abd Elaziz NY (2020). Impact of Ocimum basilicum leaves powder on immune response of chicken vaccinated against Newcastle Disease Virus. Egyptian J Agric Res.

[67] Rashidian A, Roohi P, Mehrzadi S, Ghannadi AR, Minaiyan M (2016). Protective effect of Ocimum basilicum essential oil against acetic acid–induced colitis in rats. J Evid Based Complement Alternat Med.

[68] Amrani S, Harnafi H, Gadi D, Mekhfi H, Legssyer A, Aziz M (2009). Vasorelaxant and anti-platelet aggregation effects of aqueous Ocimum basilicum extract. J Ethnopharmacol.

[69] Miraj S, Kiani S (2016). Study of pharmacological effect of Ocimum basilicum: A review. Der Pharmacia Lett.

[70] Srivastava S, Adholeya A, Conlan XA, Cahill DM (2016). Acidic potassium permanganate chemiluminescence for the determination of anti-oxidant potential in three cultivars of Ocimum basilicum. Plant Foods Human Nutr.

[71] Tavafi M, Ahmadvand H (2011). Effect of rosmarinic acid on inhibition of gentamicin induced nephrotoxicity in rats. Tissue Cell.

[72] Lee S-J, Umano K, Shibamoto T, Lee K-G (2005). Identification of volatile components in basil (Ocimum basilicum L ) and thyme leaves (Thymus vulgaris L ) and their anti-oxidant properties. Food Chem.

[73] Feng X, Xu W, Li Z, Song W, Ding J, Chen X (2019). Immunomodulatory nanosystems. Adv Sci.

[74] Keyhanmanesh R, Boskabady MH, Eslamizadeh MJ, Khamneh S, Ebrahimi MA (2010). The effect of thymoquinone, the main constituent of Nigella sativa on tracheal responsiveness and white blood cell count in lung lavage of sensitized guinea pigs. Planta Medica.

[75] Athari SS, Athari SM, Beyzay F, Movassaghi M, Mortaz E, Taghavi M (2017). Critical role of Toll-like receptors in pathophysiology of allergic asthma. Eur J Pharmacol.

[76] Boskabady MH, Keyhanmanesh R, Khamneh S, Ebrahimi MA (2011). The effect of Nigella sativa extract on tracheal responsiveness and lung inflammation in ovalbumin-sensitized guinea pigs. Clinics.

[77] Boskabady MH, Jalali S, Yahyazadeh N, Boskabady M (2016). Carvacrol attenuates serum levels of total protein, phospholipase A2 and histamine in asthmatic guinea pig. Avicenna J Phytomed.

[78] Shifren A, Witt C, Christie C, Castro M (2012). Mechanisms of remodeling in asthmatic airways. J Allergy.

[79] Rapinski M, Liu R, Saleem A, Arnason JT, Cuerrier A (2014). Environmental trends in the variation of biologically active phenolic compounds in Labrador tea, Rhododendron groenlandicum, from northern Quebec, Canada. Botany.

[80] Eftekhar N, Moghimi A, Roshan NM, Saadat S, Boskabady MH (2019). Immunomodulatory and anti-inflammatory effects of hydro-ethanolic extract of Ocimum basilicum leaves and its effect on lung pathological changes in an ovalbumin-induced rat model of asthma. BMC Complement Alternat Med.

[81] Okazaki K, Nakayama S, Kawazoe K, Takaishi Y (1998). Antiaggregant effects on human platelets of culinary herbs. Phytother Res.

[82] Yamasaki K, Nakano M, Kawahata T, Mori H, Otake t, Ueda n (1998). Anti-HIV-1 activity of herbs in Labiatae. Biol Pharm Bull.

[83] Alavinezhad A, Boskabady MH (2014). Antiinflammatory, anti-oxidant, and immunological effects of Carum copticum L and some of its constituents. Phytother Res.

[84] El-Ashram A, Afifi A, Sakr SF (2017). Effect of basil oil (Ocimum basilicum) on nonspecific immune response of Nile-tilapia (Oreochromis niloticus). Egyptian J Aquaculture.

[85] Tsai K, Lin B, Perng D, Wei J, Yu Y, Cherng J-M (2011). Immunomodulatory effects of aqueous extract of Ocimum basilicum (Linn ) and some of its constituents on human immune cells. J Med Plants Res.

[86] Gomez-Flores R, Verastegui-Rodriguez L, Quintanilla-Licea R, Tamez-Guerra P, Tamez-Guerra R, Rodriguez-Padilla C (2008). In vitro rat lymphocyte proliferation induced by Ocinum basilicum, persea americana, plantago virginica, and Rosa spp extracts. J Med Plants Res.

[87] Renzulli C, Galvano F, Pierdomenico L, Speroni E, Guerra M (2004). Effects of rosmarinic acid against aﬂatoxin B1 and ochratoxin-A-induced cell damage in a human hepatoma cell line (Hep G2). J Appl Toxicol: Int J.

[88] Dashputre NL, Naikwade NS (2010). Preliminary immunomodulatory activity of aqueous and ethanolic leaves extracts of Ocimum basilicum Linn in mice. Int J PharmTech Res.

[89] Kathirvel P, Ravi S (2012). Chemical composition of the essential oil from basil (Ocimum basilicum Linn and its in vitro cytotoxicity against HeLa and HEp-2 human cancer cell lines and NIH 3T3 mouse embryonic fibroblasts. Nat Product Res.

[90] Stanojevic LP, Marjanovic-Balaban ZR, Kalaba VD, Stanojevic JS, Cvetkovic DJ, Cakic MD (2017). Chemical composition, anti-oxidant and antimicrobial activity of basil (Ocimum basilicum L ) essential oil. J Essential Oil Bearing Plants.

[91] Rezzoug M, Bakchiche B, Gherib A, Roberta A, Kilinçarslan Ö, Mammadov R (2019). Chemical composition and bioactivity of essential oils and Ethanolic extracts of Ocimum basilicum L and Thymus algeriensis Boiss & Reut from the Algerian Saharan Atlas. BMC Complement Alternat Med.

[92] Kavoosi G, Amirghofran Z (2017). Chemical composition, radical scavenging and anti-oxidant capacity of Ocimum basilicum essential oil. J Essential oil Res.

[93] Kumar A, Shukla R, Singh P, Prakash B, Dubey NK (2011). Chemical composition of Ocimum basilicum L essential oil and its efficacy as a preservative against fungal and aflatoxin contamination of dry fruits. Int J Food Sci Technol.

[94] Tran TH, Nguyen HHH, Nguyen DC, Nguyen TQ, Tan H, Nhan LTH (2018). Optimization of microwave-assisted extraction of essential oil from Vietnamese Basil (Ocimum basilicum L ) using response surface methodology. Processes.

[95] Mehdizadeh T, Hashemzadeh M, Nazarizadeh A, Neyriz-Naghadehi M, Tat M, Ghalavand M (2016). Chemical composition and antibacterial properties of Ocimum basilicum, salvia officinalis and trachyspermum ammi essential oils alone and in combination with nisin. Res J Pharmacog.

[96] Chaaban SB, Hamdi SH, Mahjoubi K, Jemâa JMB (2019). Composition and insecticidal activity of essential oil from Ruta graveolens, Mentha pulegium and Ocimum basilicum against Ectomyelois ceratoniae Zeller and Ephestia kuehniella Zeller (Lepidoptera: Pyralidae). J Plant Dis Protect.

[97] Bobakulov K, Ozek G, Ozek T, Asilbekova DT, Abdullaev ND, Sagdullaev SS (20201). Essential oils and lipids from the flowers of two varieties of Ocimum basilicum L cultivated in Uzbekistan. J Essential Oil Res.

[98] EL MOKHTARI K, EL BROUZI A, M’hammed E, TALBI M (2020). Extraction and composition of the essential oil of Ocimum basilicum collected in Morocco. J Anal Sci Appl Biotechnol.

[99] Elsherbiny EA, EL KHATEEB AY, Azzaz NA (2016). Chemical composition and fungicidal effects of Ocimum basilicum essential oil on Bipolaris and Cochliobolus species. J Agric Sci Technol.

[100] Özcan M, Chalchat J-C (2002). Essential oil composition of Ocimum basilicum L. Czech J Food Sci.

[101] Sundararajan B, Moola AK, Vivek K, Kumari BR (2018). Formulation of nanoemulsion from leaves essential oil of Ocimum basilicum L and its antibacterial, anti-oxidant and larvicidal activities (Culex quinquefasciatus). Microbial Pathogen.

[102] Mota I, Sánchez-Sánchez J, Pedro LG, Sousa MJ (2020). Composition variation of the essential oil from Ocimum basilicum L Genovese Gigante in response to Glomus intraradices and mild water stress at different stages of growth. Biochem System Ecol.

[103] Kadan S, Saad B, Sasson Y, Zaid H (2016). In vitro evaluation of anti-diabetic activity and cytotoxicity of chemically analysed Ocimum basilicum extracts. Food Chem.

[104] Venancio AM, Onofre ASC, Lira AF, Alves PB, Blank AF, Antoniolli AR (2011). Chemical composition, acute toxicity, and antinociceptive activity of the essential oil of a plant breeding cultivar of basil (Ocimum basilicum L). Planta medica.

[105] Mousavi M, Zaiter A, Becker L, Modarressi A, Baudelaire E, Dicko A (2020). Optimisation of phytochemical characteristics and anti-oxidative properties of Foeniculum vulgare Mill seeds and Ocimum basilicum L leaves superfine powders using new parting process. Phytochem Analysis.

[106] Aye A, Jeon Y-D, Lee J-H, Bang K-S, Jin J-S (2019). Anti-inflammatory activity of ethanol extract of leaf and leaf callus of basil (Ocimum basilicum L ) on RAW 264 7 macrophage cells. Oriental Pharm Exp Med.

[107] Noor ZI, Ahmed D, Rehman HM, Qamar MT, Froeyen M, Ahmad S (2019). In vitro antidiabetic, anti-obesity and anti-oxidant analysis of Ocimum basilicum aerial biomass and in silico molecular docking simulations with alpha-amylase and lipase enzymes. Biol.

[108] Eftekhar N, Moghimi A, Boskabady MH (2018). Prophylactic effect of rosmarinic acid on tracheal responsiveness, white blood cell count and oxidative stress markers in lung lavage of sensitized rats. Pharmacol Rep.

[109] Abd El-Ghffar EA, Al-Sayed E, Shehata SM, Eldahshan OA, Efferth T (2018). The protective role of Ocimum basilicum L (Basil) against aspirin-induced gastric ulcer in mice: Impact on oxidative stress, inflammation, motor deficits and anxiety-like behavior. Food Funct.

[110] Zaveri M, Desai N, Movaliya V (2011). Effect of Ocimum basilicum on cisplatin models of acute renal failure. Adv Res Pharm boil.

[111] Rameshrad M, Salehian R, Fathiazad F, Hamedeyazdan S, Garjani M, Maleki-Dizaji N (2015). The effects of Ocimum basilicum ethanol extract on carrageenan induced paw inflammation in rats. Pharm Sci.

[112] Açıkgöz MA (2020). Establishment of cell suspension cultures of Ocimum basilicum L and enhanced production of pharmaceutical active ingredients. Ind Crops Prod.

[113] Hamad GM, Darwish AM, Abu-Serie MM, El Sohaimy SA (2017). Antimicrobial, anti-oxidant and anti-inflammatory characteristics of combination (Cassia fistula and Ocimum basilicum) extract as natural preservative to control & prevent food contamination. J Food Nutr Res.

[114] Baj T, Baryluk A, Sieniawska E (2018). Application of mixture design for optimum anti-oxidant activity of mixtures of essential oils from Ocimum basilicum L Origanum majorana L and Rosmarinus officinalis L. Ind Crops Prod.

[115] Bora KS, Arora S, Shri R (2011). Role of Ocimum basilicum L in prevention of ischemia and reperfusion-induced cerebral damage, and motor dysfunctions in mice brain. J Ethnopharmacol.

